# Influence of Aloe Vera Gel and Safe Salts on Storage Quality of Minimally Processed Carrot

**DOI:** 10.1002/fsn3.4516

**Published:** 2024-10-09

**Authors:** Swagata Ahmed, Mohammad Ali, Md. Fakhrul Hasan, Litun Ahmed Labib

**Affiliations:** ^1^ Department of Horticulture Patuakhali Science and Technology University Dumki, Patuakhali Bangladesh; ^2^ Department of Horticulture Bangabandhu Sheikh Mujibur Rahman Agricultural University Gazipur Bangladesh; ^3^ Crop Care Division Square Pharmaceuticals PLC Dhaka Bangladesh

**Keywords:** carrot, edible coating, food quality, minimally processed, postharvest treatment

## Abstract

This experiment investigated the impact of aloe vera gel and safe salts on the physical quality and physicochemical properties of minimally processed carrots during storage, aiming to determine the most effective edible coating postharvest treatment. The experiment employed a Completely Randomized Design (CRD) with three replications. Results indicated significant (*p* ≤ 0.01) effects of various treatments on the physical quality and physicochemical properties of carrots compared to untreated ones. Freshly harvested carrots were subjected to different treatments, including a control (T_1_), 30% aloe vera gel (T_2_), 1% NaCl (T_3_), and 1.5% NaHCO_3_ (T_4_), combinations thereof, such as 30% aloe vera gel with 1% NaCl (T_5_) or 1.5% NaHCO_3_ (T_6_), and 1% NaCl with 1.5% NaHCO_3_ (T_7_), and a combination of 30% aloe vera gel with 1% NaCl and 1.5% NaHCO_3_ (T_8_). Among the treatments, the combination of 30% aloe vera gel and 1% NaCl (T_5_) exhibited the most promising results after nine days of storage, with the lowest weight loss (1.19%), highest firmness (3.80 N), total soluble solids (TSS) content (8.40%), titratable acidity (0.477%), ascorbic acid content (9.02 mg/100 g FW), anthocyanin content (28.84 μg/g FW), phenol content (4.278 mg/100 g FW), and total sugar content (13.32%). This treatment effectively protected carrots from undesirable color, texture, and flavor changes during storage. The utilization of natural‐source‐based edible coatings containing health‐promoting additives presents a viable strategy to enhance both the internal and external qualities of minimally processed carrots. Overall, the edible coating comprising 30% aloe vera gel and 1% NaCl emerges as a promising approach for preserving the quality of minimally processed carrots.

## Graphical abstract

Thirty percent aloe gel + 1% NaCl coated minimally processed carrot was found to be the most effective postharvest treatment, followed by other treatment coatings in terms of minimum weight loss and higher levels of firmness, TSS, TA, vitamin C, phenol, anthocyanin, and total sugar content.

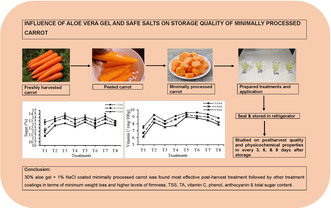

## 1 Introduction

Carrot (*Daucus carota* L.) is a biennial herbaceous species that belongs to the Apiaceae family (Carvalho et al. 2023). In spring, summer, and autumn, the carrot is one of the ancient vegetables grown in temperate regions. In Bangladesh last year (2022–23), the land under carrot cultivation was 6488.56 acres, where the total production was 35,270.61 metric tons (Yearbook of Agricultural Statistics‐2023, 2024). It is rich in carotenoids, flavonoids, vitamins, and minerals, which have been shown to have antidiabetic, low cholesterol, cardiac disease reduction, renoprotective, and wound healing properties (Dias and Dias 2014; Ikram et al. 2024).

In contemporary dietary practices, carrots are widely utilized in curries, salads, and various home‐cooked meals. This shift toward healthier, fresher, and easily prepared food options has driven increased demand for minimally processed vegetables. These products typically undergo physical modifications, including trimming, peeling, washing, chopping, or cutting, and are accompanied by packaging in sealed containers and storage at low temperatures (Perera and Smith 2013).

Despite these advancements, harvested vegetables like carrots continue to respire, consuming oxygen, and releasing carbon dioxide and water, which accelerates aging and deteriorates quality attributes such as color, taste, weight, nutrient content, and bioactive substances (Erkmen and Bozoglu 2016; Anwar, Mattoo, and Handa 2018). Minimally processed carrots are particularly susceptible to quality degradation, including the development of a whitish surface appearance and loss of nutritional and sensory qualities during storage (Condurso et al. 2020).

To address these issues, the application of edible coatings has emerged as a promising solution (Chettri, Sharma, and Mohite 2023). The main functional benefits of using edible coatings are to alter the metabolic properties of vegetable tissue by affecting respiration, prolonging storage life, firmness retention, antioxidants, etc. (Perez‐Vazquez et al. 2023). Among the polysaccharides edible coatings, starch is commonly used, and aloe vera gel is a rich source of starch having antifungal properties, preventing moisture loss and retaining firmness (Durango, Soares, and Andrade 2006; Benítez et al. 2015; Wu, Zhang, and Fan 2022). Additionally, incorporating salts like NaHCO_3_ and NaCl in coatings can further improve microbial safety and reduce water activity (Mishra, Abrol, and Dubey 2018; Farah et al. 2019). Therefore, the current research was conducted to determine the best concentration of aloe vera gel and safe salts for minimally processed carrots by studying their effects on their physical quality and physicochemical properties.

## 2 Materials and Methods

### 2.1 Experimental Location and Materials

The study was conducted in the Postharvest Laboratory, Department of Horticulture, Patuakhali Science and Technology University. Freshly harvested, mature, uniform, and disease‐free carrots were obtained from the Raypasa Khorapur Union in Barisal, Bangladesh, while the aloe vera leaves were sourced from the Horticulture Germplasm Centre, PSTU.

### 2.2 Experimental Design and Treatments

The single‐factor experiment was conducted in Completely Randomized Design (CRD) with three replications. The experiment consisted of eight treatments, viz. T_1_: Control (carrot not subjected to any treatments), T_2_: 30% solution of aloe vera gel, T_3_: 1% solution of NaCl, T_4_: 1.5% solution of NaHCO_3_, T_5_: 30% aloe vera gel + 1% NaCl, T_6_: 30% aloe vera gel + 1.5% NaHCO_3_, T_7_: 1% NaCl + 1.5% NaHCO_3_, and T_8_: 30% aloe vera gel + 1% NaCl + 1.5% NaHCO_3_.

### 2.3 Preparation of Edible Coatings and Their Application

Edible aloe vera gel coating was prepared by following the method of Ramachandran and Rao (2008). Sodium bicarbonate (NaHCO_3_) and sodium chloride (NaCl) were taken for preparation of salt treatment. Different edible coatings were prepared by mixing aloe vera gel, NaCl, and NaHCO₃ with distilled water at specified concentrations. Disease free carrots were selected, washed, manually peeled, sanitized by immersion in an 80‐ppm sodium hypochlorite solution (bleaching agent) for 2 min, and rinsed three times with distilled water. The peeled vegetables were then cut into round pieces (length 30–35 mm and diameter 10–13 mm). After this, the pieces were soaked in different coating solutions for 2 min and allowed to drip until 10 min before a second coat was applied. In the same condition, the control group has been placed under aerated water. The coated carrot pieces were dried for 1.5 h using airflow at 25°C and 45% relative humidity. Packaging materials were sterilized with UV rays for 5 min, after which the treated carrot pieces were packed and stored in a refrigerator.

### 2.4 Evaluation of Carrot Physical Quality and Physicochemical Properties

Weight loss was calculated using the formula: (Initial Weight‐Final Weight)/Initial Weight × 100 and expressed in percentage. The carrot's firmness was measured with the Digital Penetrometer (Stable MicroSystem Ltd., Surrey U) along with a measuring probe (5 mm diameter stainless steel) and expressed in newton (N). The peel color of carrot was determined by using an android application software namely “On Color Measure” (developed by Potato Tree Soft, Version 3.0). The pH of carrot was determined by using a glass electrode pH meter (GLP 21, Crison, Barcelona, and EEC). A buffer of pH 4.0 followed by pH 7.0 was calibrated to the pH meter. By using a digital refractometer from BOECO, Germany, the total soluble solids (TSS) content of carrots was estimated and expressed in percent. According to the method used by Ranganna (1977), the titratable acidity was determined and expressed in percent. Ascorbic acid content was determined according to the method of Ranganna (1986) and was expressed in mg/100 g Fw (fresh weight). The total anthocyanin content was determined by following the method of Sims and Gamon (2002) and was expressed in μg/g Fw (fresh weight). Total phenolic content was determined according to the method by Annisworth and Gillespie (2007) and expressed mg/100 g Fw (fresh weight). The sugar content of carrots was estimated by following the method Lane and Eynon (1923) and expressed in percent. All evaluations were carried out initially on day 0 and then at 3, 6, and 9 days after the treatment.

### 2.5 Statistical Analysis

The collected data were statistically analyzed by using the R package program for calculating ANOVA and the standard error (SE). The significance of the difference between treatment means was separated by Duncan's Multiple Range Test (DMRT) at the 1% level of probability.

## 3 Results And Discussion

### 3.1 Composition of the Fresh Carrot

The firmness, pH, total soluble solids (TSS), titratable acidity (TA), anthocyanin content, ascorbic acid, phenol content, and total sugar percentage are presented below:

**Table:** 

SL. no.	Constituents (parameters)	Carrot
1.	Firmness	4.03%
2.	pH	6.52
3.	Total soluble solids (TSS)	8.50 (%Brix)
4.	Titratable acidity (TA)	0.326%
5.	Anthocyanin	29.54 μg/g FW
6.	Ascorbic acid (Vit‐C)	9.82 mg/100 g FW
7.	Phenol content	5.02 mg/100 g FW
8.	Total sugar	14.57%

### 3.2 Weight Losses of Carrot During Storage

Carrots have a significant difference (*p* ≤ 0.01) in weight loss due to different edible coating treatments (Table 1). At the 3, 6, and 9 days after storage (DAS), control (T_1_) showed the highest weight loss of carrots (1.63%, 2.31%, and 3.24%), whereas treatment T_5_ showed the lowest (0.69%, 0.94%, and 1.19%). Weight loss is a crucial measure of postharvest quality, largely driven by water loss through respiration and transpiration (Islam et al. 2019). Table 1 illustrates that control samples had higher weight loss compared to those with edible coatings, with treatment T_5_ (30% aloe vera gel with 1% NaCl) showing the lowest loss. The aloe vera gel coating effectively served as a moisture barrier, controlling the permeability of water vapor, oxygen, and carbon dioxide (Farina et al. 2020). Additionally, adding NaCl coating further reduced weight loss by minimizing respiration, water loss, and fungal activity (Palou et al. 2007). These observations support the findings of Farooq et al. (2023), which indicated that similar coatings significantly lower weight loss in fresh produce.

**TABLE 1:** Effect of different edible coating treatments on weight loss and firmness of carrot at different days after storage.

Treatments	Weight loss (%)			Firmness (N)		
	3 DAS	6 DAS	9 DAS	3 DAS	6 DAS	9 DAS
T_1_	1.63 a	2.31 a	3.24 a	3.23 bc	3.07 c	2.82 e
T_2_	0.87 e	1.16 d	2.03 b	3.53 bc	3.60 ab	3.20 cde
T_3_	0.89 d	1.27 b	1.74 c	3.23 bc	3.43 b	3.57 abc
T_4_	0.98 c	1.22 c	1.38 f	3.27 bc	3.33 bc	3.17 de
T_5_	0.69 h	0.94 g	1.19 g	3.60 b	3.63 ab	3.80 a
T_6_	0.81 f	0.99 f	1.40 f	4.10 a	3.83 a	3.63 ab
T_7_	0.78 g	1.13 e	1.44 e	3.40 bc	3.90 a	3.77 a
T_8_	1.03 b	1.23 c	1.51 d	3.17 c	3.37 bc	3.33 bcd
Level of Sig.	**	**	**	**	**	**
CV (%)	1.17	0.93	0.79	7.03	4.72	6.23
LSD at 1%	0.019	0.021	0.024	0.421	0.289	0.370

*Note:* Here, T_1_ = Control, T_2_ = 30% aloe vera gel, T_3_ = 1% NaCl, T_4_ = 1.5% NaHCO_3_, T_5_ = 30% aloe vera gel + 1% NaCl, T_6_ = 30% aloe vera gel + 1.5% NaHCO_3_, T_7_ = 1% NaCl + 1.5% NaHCO_3_, T_8_ = 30% aloe vera gel + 1% NaCl + 1.5% NaHCO_3_.

Abbreviation: DAS, days after storage.

** Significant at 1% level of probability. In a column, values having different letters differ significantly at 1% level of probability analyzed by DMRT.

### 3.3 Loss of Firmness During the Storage

A considerable (*p* ≤ 0.01) difference has been noted in carrot firmness, due to the effects of different treatments (Table 1). In the 3 DAS, the highest firmness of carrot (4.10 N) was observed from treatment T_6_, and the lowest (3.17 N) was observed from treatment T_8_. In the 6 DAS, the highest firmness of carrot (3.90 N) was observed from treatment T_7_, which was statistically similar to treatment T_6_, whereas the lowest firmness of carrot (3.07 N) was observed from treatment T_1_. At the 9 DAS, the highest firmness of carrot (3.80 N) was found from treatment T_5_, which was statistically similar to T_7_, whereas the lowest (2.82 N) was recorded from the T_1_ treatment. The loss of firmness over storage periods is a typical outcome of the fruit's metabolic and physiological processes, particularly due to the action of the polygalacturonase enzyme, which contributes to softening (Paniagua et al. 2020). Similar observations have been reported for table grapes and cherries coated with aloe vera gel, which showed delayed firmness loss and color changes (Serrano et al. 2006). NaCl may reduce fruit softening by inhibiting the activity of cell wall‐degrading enzymes. On the contrary, NaHCO_3_ decreased the firmness, indicating increased toxicity and a failure to preserve cell turgidity and peel thickness. The coatings effectively slow respiration rates and reduce the breakdown of insoluble protopectins into pectin acid and pectin, thereby decelerating the ripening process and preserving firmness (Pietrosanto et al. 2022).

### 3.4 Changes in External Peel Color During Storage

The values of red (R), green (G), and blue (B) were measured to monitor changes in the outer color of carrots. Values are presented in Table 2.

**TABLE 2:** Effect of different edible coating treatments on the external peel color of carrot on different days after storage.

Treatments	Red			Green			Blue		
	3 DAS	6 DAS	9 DAS	3 DAS	6 DAS	9 DAS	3 DAS	6 DAS	9 DAS
T_1_	167.67 d	156.00 c	144.00 f	86.33 b	72.33 f	64.67 fg	34.00 bcd	31.00 cd	27.00 d
T_2_	182.33 a	187.00 a	184.33 a	91.00 a	105.67 b	87.67 a	36.67 bc	44.00 a	46.00 a
T_3_	163.67 e	158.00 c	152.00 e	66.67 e	58.00 h	65.33 f	27.33 e	28.33 d	29.00 cd
T_4_	157.67 f	157.67 c	172.67 b	72.33 d	92.00 c	79.33 c	41.67 a	39.67 b	37.67 b
T_5_	175.33 b	184.00 ab	166.67 c	90.33 a	108.67 a	84.33 b	33.33 cd	43.67 a	37.00 b
T_6_	171.67 c	158.33 c	157.33 d	76.00 c	65.00 g	62.00 g	32.00 d	31.00 cd	30.67 c
T_7_	154.33 g	152.00 d	168.00 c	56.00 f	75.33 e	72.00 e	24.00 e	44.33 a	36.00 b
T_8_	174.00 b	182.00 b	175.67 b	84.67 b	84.00 d	75.00 d	37.33 b	32.33 c	35.00 b
Level of Sig.	**	**	**	**	**	**	**	**	**
CV (%)	0.66	1.35	1.49	2.32	1.69	2.19	5.92	4.81	4.62
LSD at 1%	1.928	3.911	4.281	3.148	2.424	2.809	3.432	3.083	2.798

*Note:* Here, T_1_ = Control, T_2_ = 30% aloe vera gel, T_3_ = 1% NaCl, T_4_ = 1.5% NaHCO_3_, T_5_ = 30% aloe vera gel + 1% NaCl, T_6_ = 30% aloe vera gel + 1.5% NaHCO_3_, T_7_ = 1% NaCl + 1.5% NaHCO_3_, T_8_ = 30% aloe vera gel + 1% NaCl + 1.5% NaHCO_3_.

Abbreviation: DAS, days after storage.

** Significant at 1% level of probability. In a column, values having different letters differ significantly at 1% level of probability analyzed by DMRT.

#### 3.4.1 Red

At the 3, 6, and 9 DAS, the highest red color (182.33, 187, and 184.33) of carrot was measured from treatment T_2_, whereas at 3 and 6 DAS the lowest (154.33 and 152) red color was recorded from T_7_ treatment, and at 9 DAS the lowest (144) was recorded from T_1_ treatment.

#### 3.4.2 Green

At the 3 DAS, the highest green color (91) was found from treatment T_2_, whereas the lowest (56) was recorded from T_7_ treatment. At the 6 DAS, the maximum green color (108.67) was observed from T_5_, whereas the minimum (58) from T_3_ treatment. And at the 9 DAS, the highest green color (87.67) was measured from T_2_, whereas the lowest (62) was measured from T_6_ treatment.

#### 3.4.3 Blue

At the 3 DAS, the highest blue color (41.67) was measured from treatment T_4_, whereas the lowest (24) was measured from the T_7_ treatment. At the 6 DAS, the maximum blue color (44.33) was observed from T_7_, which was statistically similar to T_2_ and T_5_ treatments and a minimum (28.33) was recorded from T_3_ treatment. At the 9 DAS, the highest blue color (46) was found from T_2_, whereas the lowest (27) was found from the T_1_ treatment.

The results indicate that edible coating treatments significantly (*p* ≤ 0.01) affect the external peel color of carrots, with differences observed based on both the type of coating and the duration of storage (Table 2). Aloe gel alone (T_2_) and its combination with other treatments (T_5_ and T_8_) were notably effective in preserving the red, green, and blue color components of the carrot peels throughout the storage period. This suggests that aloe gel with NaCl and NaHCO_3_ has substantial potential in maintaining the visual appeal of carrots. The antifungal and antimicrobial properties of salt may contribute to color retention (Serna‐Escolano et al. 2023). Combination treatments, especially T_8_, which included aloe gel, NaCl, and NaHCO_3_, demonstrated enhanced color retention, suggesting a synergistic effect that warrants further investigation.

### 3.5 Changes in pH of Carrot During Storage

**FIGURE 1:**
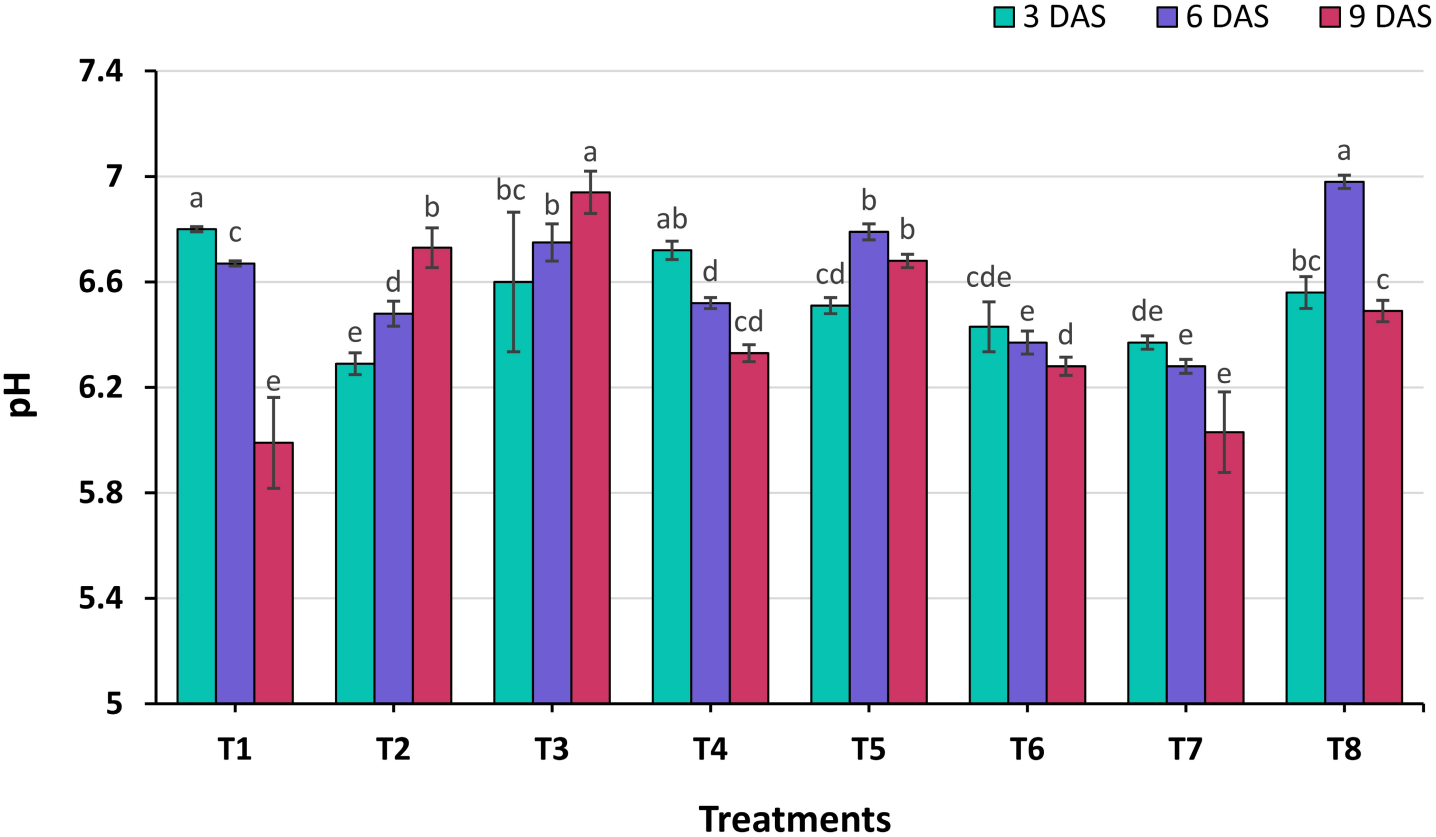
Effect of different edible coating treatments on pH of carrot at different days after storage. Here, DAS, days after storage. The vertical bars represent the standard error. Different letters indicate significant differences at the 1% level of probability, as determined by DMRT.

Carrots had a significant variation (*p* ≤ 0.01) in pH at each of the treatments in Figure 1. At the 3 DAS, the highest pH (6.80) was observed from treatment T_1_, whereas the lowest (6.29) was observed from the T_2_ treatment. At the 6 DAS, the maximum pH (6.98) was recorded from T_8_, whereas the minimum (6.28) was recorded from T_7_ treatment. And at the 9 DAS, the maximum pH (6.94) was found from T_3_, whereas the minimum (5.99) was found from T_1_. A decrease in pH values can be linked to an increase in titratable acidity, which may result from a reduced respiration rate (Jiang and Li 2001). The control (T_1_) exhibited a gradual decline in pH over time, suggesting potential spoilage or an increase in acidity. In contrast, T_5_ showed relatively stable pH levels, indicating effective buffering properties. Salts change fruit peel pH and inhibit early‐stage cell wall‐degrading enzymes produced by pathogens, and increases in salt concentration result in decreases of pH (Albersheim et al. 2011; Karmaker et al. 2024). Previous studies have reported that tomato fruits treated with aloe vera maintained pH levels better compared to untreated fruits (Athmaselvi, Sumitha, and Revathy 2013). Similar findings were observed in avocado fruits, as noted by Maftoonazad and Ramaswamy (2005).

### 3.6 Changes in Total Soluble Solids (TSS) Concentration During Storage

With the use of various treatments, there was a considerable change (*p* ≤ 0.01) in the total soluble solid concentration of carrot (Table 3). At the 3 DAS, the highest TSS (8.87%) was measured from treatment T_5_, and the lowest (6.90%) was measured from the T_4_ treatment. At the 6 DAS, the highest TSS (9.17%) was observed from T_6_, which was statistically similar to T_5_ treatment, whereas the lowest (7.60%) was observed from T_1_ treatment. And at the 9 DAS, the maximum TSS (8.40%) was recorded from T_5_, which was statistically similar to the T_3_ treatment, whereas the minimum (6.90%) was recorded from the T_1_ treatment. The higher TSS values observed in aloe gel treatments (T_2_, T_5_, and T_6_) suggest that these coatings effectively slow down the respiration rate and reduce biochemical changes, thereby preserving carbohydrate content. This is consistent with the observation that TSS tends to increase initially due to the hydrolysis of starches into sugars, followed by a decrease as sugars are converted into organic acids or other compounds (Javed et al. 2017). Previous studies have highlighted similar findings, where aloe vera gel and other edible coatings, such as honey, effectively slowed down biochemical changes and preserved TSS in fruits and vegetables (Kuwar, Sharma, and Tadapaneni 2015).

**TABLE 3:** Effect of different edible coating treatments on total soluble solids (TSS) percentage and titratable acidity (%) of carrot on different days after storage.

Treatments	TSS (%)			Titratable acidity (%)		
	3 DAS	6 DAS	9 DAS	3 DAS	6 DAS	9 DAS
T_1_	7.83 c	7.60 c	6.90 e	0.174 f	0.193 f	0.256 f
T_2_	8.70 ab	8.47 b	7.87 bc	0.265 e	0.196 f	0.210 g
T_3_	7.33 d	7.73 c	8.37 a	0.332 b	0.257 e	0.198 h
T_4_	6.90 e	7.77 c	7.27 d	0.286 d	0.384 a	0.436 b
T_5_	8.87 a	9.00 a	8.40 a	0.383 a	0.296 c	0.477 a
T_6_	8.77 a	9.17 a	7.77 c	0.324 c	0.342 b	0.384 c
T_7_	7.00 e	7.70 c	7.63 c	0.284 d	0.292 c	0.343 d
T_8_	8.40 b	8.30 b	8.13 ab	0.320 c	0.268 d	0.295 e
Level of Sig.	**	**	**	**	**	**
CV (%)	2.34	3.10	2.32	1.26	1.43	1.61
LSD at 1%	0.325	0.444	0.314	0.006	0.006	0.006

*Note:* Here, T_1_ = Control, T_2_ = 30% aloe vera gel, T_3_ = 1% NaCl, T_4_ = 1.5% NaHCO_3_, T_5_ = 30% aloe vera gel + 1% NaCl, T_6_ = 30% aloe vera gel + 1.5% NaHCO_3_, T_7_ = 1% NaCl + 1.5% NaHCO_3_, T_8_ = 30% aloe vera gel + 1% NaCl + 1.5% NaHCO_3_.

Abbreviation: DAS, days after storage.

** Significant at 1% level of probability. In a column, values having different letters differ significantly at 1% level of probability analyzed by DMRT.

### 3.7 Changes in Titratable Acidity (TA) During Storage

The titratable acidity (TA) of carrots varied significantly (*p* ≤ 0.01) due to the application of different treatments (Table 3). At the 3 DAS, the maximum TA (0.383%) was measured from treatment T_5_, whereas the lowest (0.174%) was measured from the T_1_ treatment. At the 6 DAS, the highest TA (0.384%) was observed from treatment T_4_, whereas the lowest (0.193%) was observed from the T_1_ treatment. And at the 9 DAS, the maximum TA (0.477%) was found from treatment T_5_, whereas the minimum (0.198%) was found from the T_3_ treatment. The results reveal that edible coatings significantly impact the titratable acidity (TA) of carrots during storage. The aloe vera gel treatments (T_2_) and their combinations (T_5_) effectively delayed the decrease in TA, indicating better preservation of organic acids. This preservation is likely due to reduced respiration rates and decreased oxygen diffusion facilitated by the coatings. Similar findings have been reported for other fruits, where coatings reduced the loss of ascorbic acid and maintained acidity levels by limiting respiration and oxidation (Youssef and Roberto 2014; Martinez‐Espla et al. 2018).

### 3.8 Changes in Ascorbic Acid Content During Storage

**FIGURE 2:**
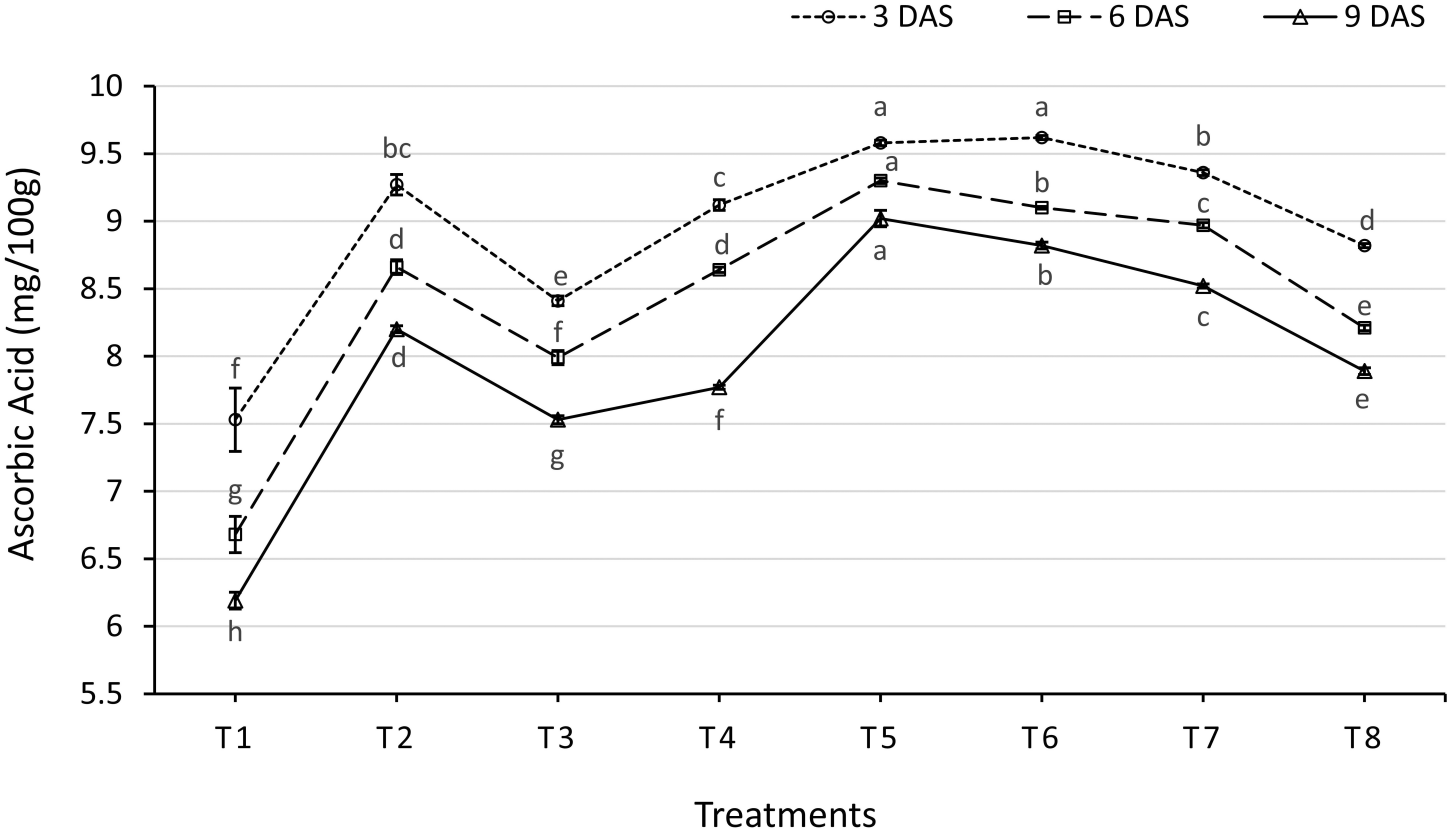
Effect of different edible coating treatments on ascorbic acid content of carrot at different days after storage. Here, DAS, days after storage. The vertical bars represent the standard error. Different letters indicate significant differences at the 1% level of probability, as determined by DMRT.

Concerning the ascorbic acid content in carrots, there has been a very wide difference between various methods of food coating (Figure 2). At the 3 DAS, the highest ascorbic acid (9.62 mg/100 g FW) was recorded from treatment T_6_, which was statistically similar to T_5_ (9.58 mg/100 g FW) treatment, whereas the lowest (7.53 mg/100 g FW) was recorded from the T_1_ treatment. At the 6 DAS, the highest ascorbic acid (9.30 mg/100 g FW) was observed from T_5_, whereas the lowest (6.68 mg/100 g FW) was observed from T_1_ treatment. And at the 9 DAS, the maximum ascorbic acid (9.02 mg/100 g FW) was found from T_5_, whereas the minimum (6.19 mg/100 g FW) was found from the T_1_ treatment. Aloe vera gel and safe salt treatments (T_2_, T_5_, and T_6_) effectively reduced the decline in ascorbic acid content compared to the control, which experienced a substantial decrease over time. Aloe vera coatings appear to mitigate the oxidation of ascorbic acid, as previously reported (Mditshwa et al. 2017). This preservation is likely due to aloe vera gel's role with NaCl and NaHCO_3_ in reducing oxidative stress and delaying the conversion of ascorbic acid to dehydroascorbic acid and further degradation. These findings align with previous research that demonstrated the efficacy of aloe vera and other edible coatings in maintaining ascorbic acid levels by limiting oxidation and extending shelf life (Nasution, Ye, and Hamzah 2015; Paul et al. 2023).

### 3.9 Changes in Anthocyanin Content During Storage

Due to the use of different treatments, the anthocyanin content of carrots has changed significantly (*p* ≤ 0.01). Table 4 illustrates that, at the 3 DAS, the highest anthocyanin content (29.92 μg/g FW) was recorded from treatment T_6_, whereas the lowest (28.19 μg/g FW) was recorded from the T_1_ treatment. At the 6 and 9 DAS, the maximum anthocyanin content (29.17 and 28.84 μg/g FW) was observed from T_5_, whereas the minimum (27.16 and 25.49 μg/g FW) was observed from T_1_ treatment. The combination of 30% aloe vera gel with 1% NaCl (T_5_) and 30% aloe vera gel with 1.5% NaHCO3 (T_6_) consistently resulted in the highest anthocyanin levels across all storage periods, indicating a delay in the ripening process. In contrast, the control and treatments with single additives (T_1_, T_3_, T_4_, and T_7_) showed lower anthocyanin levels, with significant decreases observed over time. The treatment with all three additives (T_8_) did not perform as well as T_5_ and T_6_. These results are consistent with previous studies indicating that edible coatings can effectively slow ripening and preserve anthocyanin content (Tang, He, and Fan 2023).

**TABLE 4:** Effect of different edible coating treatments on anthocyanin content and phenol of carrot at different days after storage.

Treatments	Anthocyanin (μg/g FW)			Phenol (mg/100 g FW)		
	3 DAS	6 DAS	9 DAS	3 DAS	6 DAS	9 DAS
T_1_	28.19 h	27.16 f	25.49 h	4.190 cd	4.013 d	3.822 c
T_2_	29.78 c	29.02 b	28.46 c	5.115 a	4.677 a	4.011 b
T_3_	28.57 g	27.91 e	27.33 f	4.323 c	4.010 d	3.623 d
T_4_	29.68 d	28.88 c	28.13 d	4.657 b	4.417 b	4.145 ab
T_5_	29.86 b	29.17 a	28.84 a	5.033 a	4.731 a	4.278 a
T_6_	29.92 a	29.06 b	28.79 b	5.027 a	4.697 a	4.282 a
T_7_	28.76 f	27.84 e	26.92 g	4.057 d	4.230 c	4.082 b
T_8_	28.87 e	28.14 d	27.41 e	4.547 b	4.325 bc	4.033 b
Level of Sig.	**	**	**	**	**	**
CV (%)	0.102	0.168	0.088	2.51	1.57	2.13
LSD at 1%	0.052	0.083	0.042	0.202	0.120	0.150

*Note:* Here, T_1_ = Control, T_2_ = 30% aloe vera gel, T_3_ = 1% NaCl, T_4_ = 1.5% NaHCO_3_, T_5_ = 30% aloe vera gel + 1% NaCl, T_6_ = 30% aloe vera gel + 1.5% NaHCO_3_, T_7_ = 1% NaCl + 1.5% NaHCO_3_, T_8_ = 30% aloe vera gel + 1% NaCl + 1.5% NaHCO_3_.

Abbreviation: DAS, days after storage.

** Significant at 1% level of probability. In a column, values having different letters differ significantly at 1% level of probability analyzed by DMRT.

### 3.10 Changes in Phenolic Content During Storage

Significant differences (*p* ≤ 0.01) in the phenol content of carrots were observed over a number of days following storage (Table 4). At the 3 DAS, the maximum phenol content (5.115 mg/100 g FW) was recorded from treatment T_2_, which was statistically similar to T_5_ and T_6_ treatments, whereas the minimum (4.057 mg/100 g FW) was recorded from the T_7_ treatment. At the 6 DAS, the highest phenol content (4.731 mg/100 g FW) was observed from T_5_, whereas the minimum (4.010 mg/100 g FW) was observed from T_3_ treatment, which was statistically similar to T_1_ treatment. And at the 9 DAS, the maximum phenol content (4.282 mg/100 g FW) was found from T_6_, which was statistically similar to T_5_, whereas the minimum (3.623 mg/100 g FW) was found from the T_3_ treatment. The observed increase in phenolic content with aloe vera gel treatments is consistent with findings by Davila‐Avina et al. (2014), who noted that edible coatings like aloe vera gel can influence the synthesis of secondary metabolites. This effect may be attributed to the reduction of oxidative stress and altered metabolic pathways, as suggested by Wang et al. (2022), who reported that stress‐activated enzymes like phenylalanine ammonia‐lyase (PAL) can enhance phenolic synthesis under controlled conditions. The significant decline in phenolic content in control carro (T_1_) supports previous research indicating that uncoated fruits undergo more pronounced degradation of phenolic compounds during storage (Kushwaha et al. 2021).

### 3.11 Changes in Total Sugar Content During Storage

**FIGURE 3:**
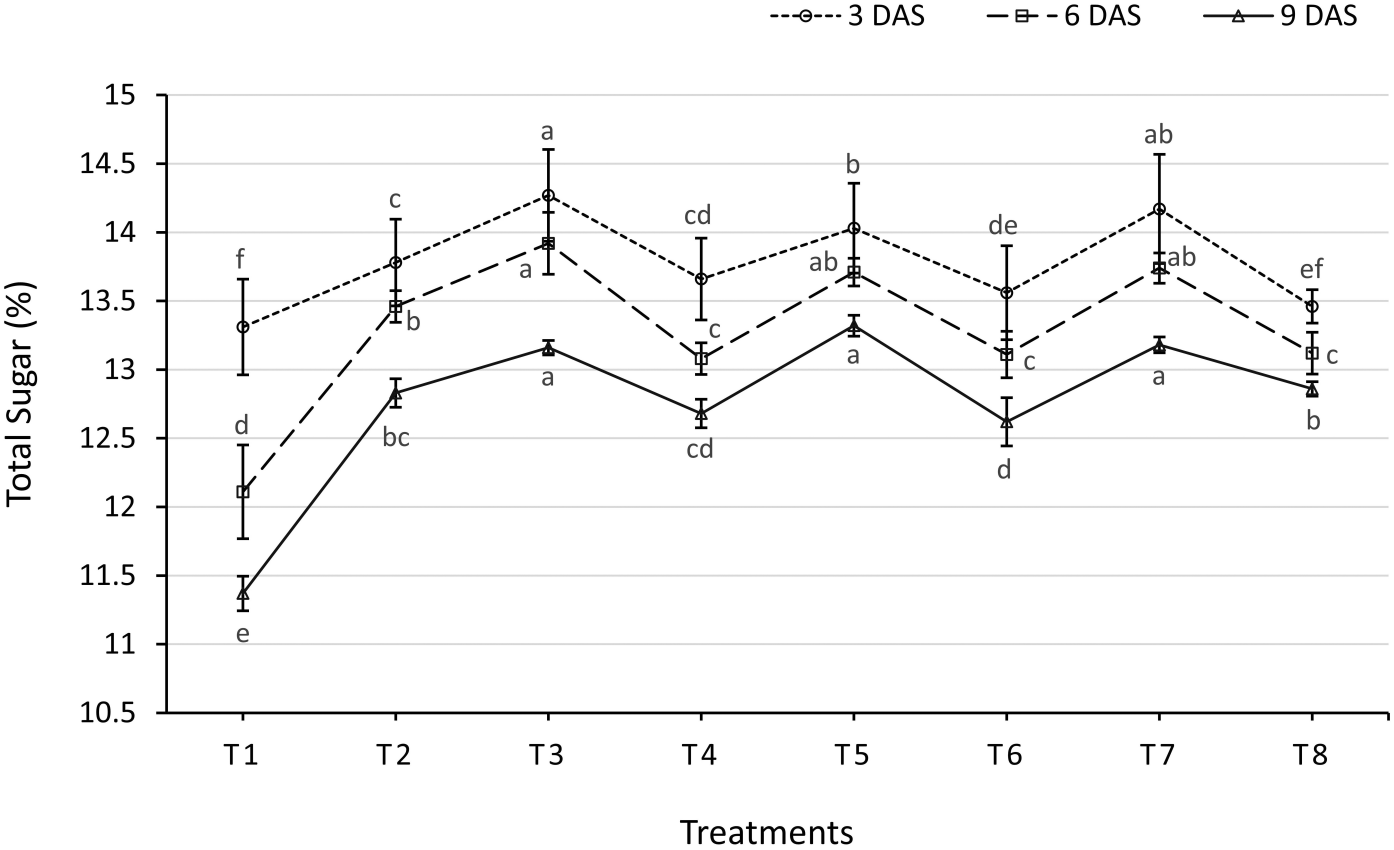
Effect of different edible coating treatments on total sugar content of carrot at different days after storage. Here, DAS, days after storage. The vertical bars represent the standard error. Different letters indicate significant differences at the 1% level of probability, as determined by DMRT.

A considerable difference between the different treatment types has been noted during storage (Figure 3). At the 3 DAS, the highest sugar content (14.27%) was found from treatment T_3_, whereas the lowest (13.31%) was found from the T_1_ treatment. At the 6 DAS, the maximum sugar content (13.92%) was observed from T_3_, which was statistically almost near to T_7_ (13.74%) and T_5_ (13.71%), whereas the minimum (12.11%) was observed from T_1_ treatment. And at the 9 DAS, the highest sugar content (13.32%) was recorded from T_5_, whereas the minimum (11.37%) was recorded from the T_1_ treatment. The results indicate that the treatments have a significant impact (*p* ≤ 0.01) on total sugar content throughout the storage period. Treatment 3 (1% NaCl) consistently maintained the highest total sugar percentages on all days. In contrast, the control (T1) showed the lowest sugar retention, especially evident at 9 days of storage. Treatment 5 (30% aloe Vera Gel + 1% NaCl) also demonstrated a considerable sugar retention effect, suggesting that aloe Vera Gel and NaCl contribute to sugar preservation and these fingdings is supported by Ullah et al. (2017).

### 3.12 Correlation Coefficient Analysis

**FIGURE 4:**
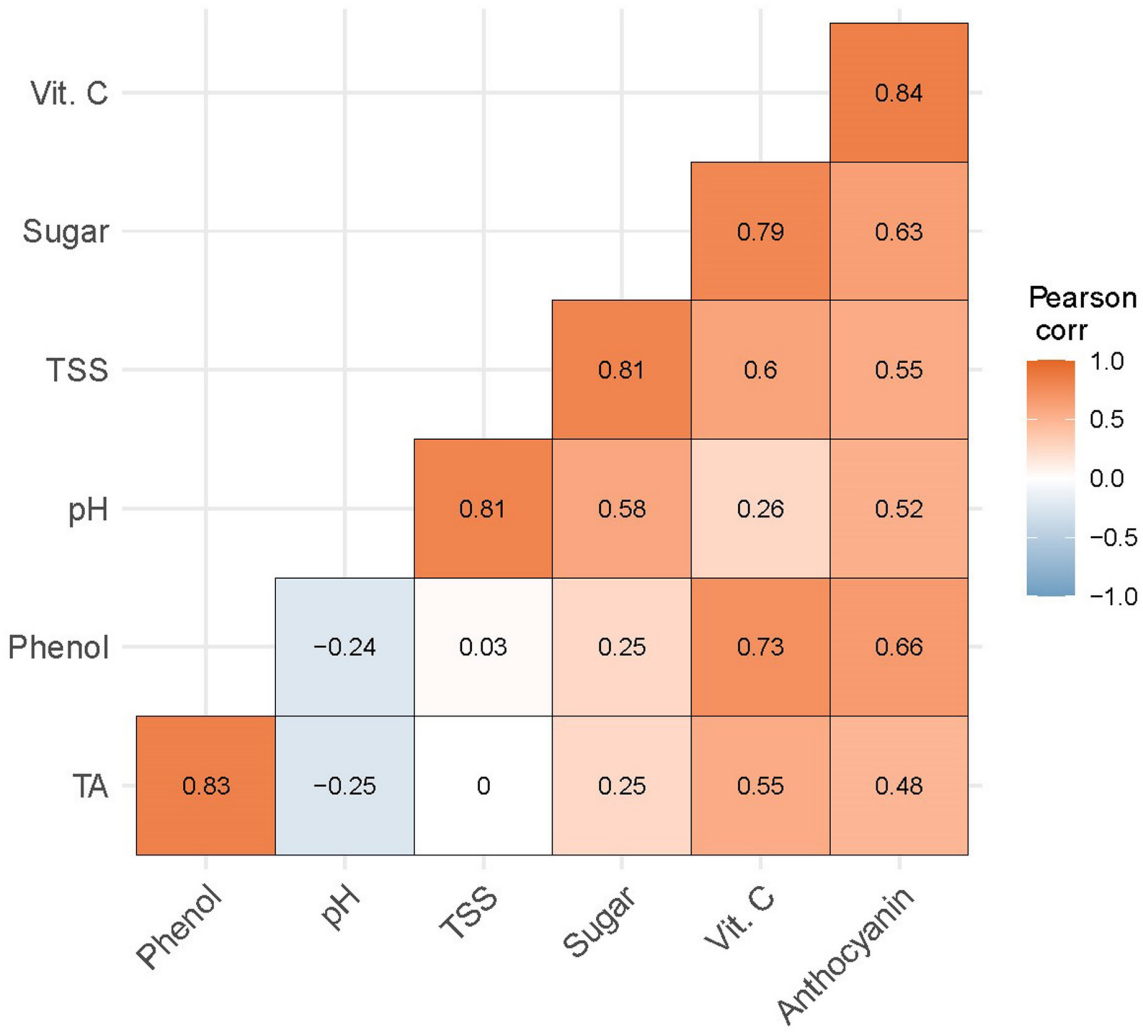
Correlation coefficient analysis of pH, TSS, TA, ascorbic acid, anthocyanin, phenol, and total sugar in minimally processed carrots after 9 days of storage.

Figure 4 illustrates the Pearson correlation coefficients among various variables related to carrot quality, with values ranging from negative to positive correlations, as indicated by a color gradient from red to purple. White color cells were considered a nonsignificant (zero) relationship among the variables at the 5% level of significance. The red color square (0.5–1.0) denotes the stronger correlation between the two respective variables. From the total variables, there was a strong negative correlation (*R* ^2^ = −0.25, −0.24) between the pH and titratable acidity (TA) and phenol content, respectively, which means that an increase in pH brings about a decrease in these parameter's value, whereas pH had a significant positive correlation with total soluble solids (TSS), total sugar, and anthocyanin content (*R* ^2^ = 0.81, 0.58, 0.52) and it showed insignificant (no relationship) associations with ascorbic acid (*R* ^2^ = 0.26). Moreover, TSS had highly significant positive total sugar, ascorbic acid, and anthocyanin content (*R* ^2^ = 0.81, 0.60, 0.55). Furthermore, TA had significant positive ascorbic acid and anthocyanin content (*R* ^2^ = 0.55, 0.48) while it showed insignificant (no relationship) associations with TSS and total sugar (*R* ^2^ = 0.00, 0.25). In addition, phenol had significant positive correlation with ascorbic acid and anthocyanin content (*R* ^2^ = 0.73, 0.66) while it showed insignificant (no relationship) associations with TSS and total sugar (*R* ^2^ = 0.03, 0.25). Total sugar had a strong correlation with ascorbic acid (*R* ^2^ = 0.79) and ascorbic acid had a significant correlation coefficients with anthocyanin (*R* ^2^ = 0.84), respectively. This correlation analysis highlights the complex interrelationships among key quality parameters of carrots, emphasizing the influence of pH on other attributes and revealing the strong associations between TSS, total sugar, and ascorbic acid with anthocyanin content.

## 4 Conclusion

The study evaluates the effectiveness of various postharvest treatments on maintaining the quality of minimally processed carrots during storage in the refrigerator. Results demonstrate that coating minimally processed carrots with 30% aloe vera gel and 1% NaCl significantly enhances consumer acceptability and reduces spoilage loss. This treatment extends the shelf life of carrots up to 9 days with minimal nutritional deterioration. It improves appearance, firmness, and overall quality, resulting in reduced weight loss and elevated levels of firmness, total soluble solids (TSS), titratable acidity (TA), ascorbic acid, anthocyanin, phenol, and total sugar content. Furthermore, it effectively prevents undesirable color, texture, and flavor changes during storage. The use of natural‐source‐based edible coatings with health‐promoting additives enhances both internal and external carrot qualities. The findings highlight the potential of 30% aloe vera gel and 1% NaCl coating for maintaining minimally processed carrot quality during storage. Recommendations include further validation through larger market‐simulated experiments and potential extension to other root vegetables.

## Author Contributions

**Swagata Ahmed:** conceptualization (lead), data curation (equal), formal analysis (equal), investigation (lead), methodology (equal), software (supporting), supervision (equal), validation (equal), visualization (equal), writing – original draft (equal), writing – review and editing (equal). **Mohammad Ali:** conceptualization (equal), funding acquisition (lead), investigation (equal), methodology (supporting), resources (equal), supervision (lead), validation (equal). **Md.** **Fakhrul Hasan:** conceptualization (equal), investigation (equal), methodology (lead), resources (lead), supervision (equal), validation (equal), writing – review and editing (supporting). **Litun Ahmed Labib:** conceptualization (equal), data curation (lead), formal analysis (lead), investigation (equal), methodology (equal), software (lead), supervision (equal), validation (lead), visualization (lead), writing – original draft (lead), writing – review and editing (lead).

## Ethics Statement

The authors have nothing to report.

## Conflicts of Interest

The authors declare no conflicts of interest.

## Data Availability

Data presented in this study are available from the corresponding author upon request.

## References

[fsn34516-bib-0001] Albersheim, P. , A. Darvill , K. Roberts , R. Sederoff , and A. Staehelin . 2011. “Cell Walls and Plant–Microbe Interactions.” In Plant Cell Walls: From Chemistry to Biology; Garland Science, 319–363. New York, NY: Taylor & Francis Group LLC.

[fsn34516-bib-0002] Annisworth, E. A. , and K. Gillespie . 2007. “Estimation of Total Phenolic Content and Other Oxidation Substrates in Plant Tissues Using Folin–Ciocalteu Reagent.” Nature Protocols 2: 875–877. 10.1038/nprot.2007.102. 17446889

[fsn34516-bib-0003] Anwar, R. , A. K. Mattoo , and A. K. Handa . 2018. “Ripening and Senescence of Fleshy Fruits.” In Postharvest Biology and Nanotechnology of Fruits, Vegetables and Flowers, 15–51. New Jersey: Wiley Online Library. 10.1002/9781119289470.CH2.

[fsn34516-bib-0004] Athmaselvi, K. A. , P. Sumitha , and B. Revathy . 2013. “Development of *Aloe Vera* Based Edible Coating for Tomato.” International Agrophysics 27, no. 4: 369–375. 10.2478/intag-2013-0006.

[fsn34516-bib-0005] Benítez, S. , I. Achaerandio , M. Pujolà , and F. Sepulcre . 2015. “Aloe Vera as an Alternative to Traditional Edible Coatings Used in Fresh‐Cut Fruits: A Case of Study With Kiwifruit Slices.” LWT ‐ Food Science and Technology 61, no. 1: 184–193.

[fsn34516-bib-0006] Carvalho, G. R. , K. C. Santos , J. S. Guedes , B. S. Bitencourt , M. L. Rojas , and P. E. D. Augusto . 2023. “Drying of Roots and Tubers.” In Drying Technology in Food Processing: Unit Operations and Processing Equipment in the Food Industry, 587–628. Woodhead Publishing. 10.1016/B978-0-12-819895-7.00018-3.

[fsn34516-bib-0007] Chettri, S. , N. Sharma , and A. M. Mohite . 2023. “Edible Coatings and Films for Shelf‐Life Extension of Fruit and Vegetables.” Biomaterials Advances 154: 213632. 10.1016/J.BIOADV.2023.213632. 37742558

[fsn34516-bib-0008] Condurso, C. , F. Cincotta , G. Tripodi , M. Merlino , F. Giarratana , and A. Verzera . 2020. “A New Approach for the Shelf‐Life Definition of Minimally Processed Carrots.” Postharvest Biology and Technology 163: 111138. 10.1016/J.POSTHARVBIO.2020.111138.

[fsn34516-bib-0009] Davila‐Avina, J. E. , J. A. Villa‐Rodríguez , M. A. Villegas‐Ochoa , et al. 2014. “Effect of Edible Coatings on Bioactive Compounds and Antioxidant Capacity of Tomatoes at Different Maturity Stages.” Journal of Food Science and Technology 51: 2706–2712. 10.1007/s13197-012-0771-3. 25328215 PMC4190203

[fsn34516-bib-0010] Dias, J. C. d. S. , and J. C. d. S. Dias . 2014. “Nutritional and Health Benefits of Carrots and Their Seed Extracts.” Food and Nutrition Sciences 5, no. 22: 2147–2156. 10.4236/FNS.2014.522227.

[fsn34516-bib-0011] Durango, A. M. , N. F. F. Soares , and N. J. Andrade . 2006. “Microbiological Evaluation of an Edible Antimicrobial Coating on Minimally Processed Carrots.” Food Control 17, no. 5: 336–341. 10.1016/J.FOODCONT.2004.10.024.

[fsn34516-bib-0012] Erkmen, O. , and T. F. Bozoglu . 2016. “Food Preservation by Combination of Techniques (Hurdle Technology).” In Food Microbiology: Principles Into Practice, edited by O. Erkmen and T. F. Bozoglu , vol. 1, 1–458. John Wiley & Sons, Ltd. 10.1002/9781119237860.

[fsn34516-bib-0013] Farah, I. O. , W. O. Lyons , Z. Arslan , G. Miller , H. Benghuzzi , and P. B. Tchounwou . 2019. “Sodium Bicarbonate Remediation of Anthropogenic Contamination of Water at the Gbnerr in Mississippi.” Biomedical Sciences Instrumentation 55, no. 2: 497–504. /pmc/articles/PMC6785824. 31602051 PMC6785824

[fsn34516-bib-0014] Farina, V. , R. Passafiume , I. Tinebra , E. Palazzolo , and G. Sortino . 2020. “Use of Aloe Vera Gel‐Based Edible Coating With Natural Anti‐Browning and Anti‐Oxidant Additives to Improve Post‐Harvest Quality of Fresh‐Cut ‘Fuji’ Apple.” Agronomy 10, no. 4: 515. 10.3390/agronomy10040515.

[fsn34516-bib-0015] Farooq, A. , B. Niaz , F. Saeed , et al. 2023. “Exploring the Potential of Aloe Vera Gel‐Based Coating for Shelf Life Extension and Quality Preservation of Tomato.” International Journal of Food Properties 26, no. 2: 2909–2923. 10.1080/10942912.2023.2263661.

[fsn34516-bib-0016] Ikram, A. , A. Rasheed , A. Ahmad Khan , et al. 2024. “Exploring the Health Benefits and Utility of Carrots and Carrot Pomace: A Systematic Review.” International Journal of Food Properties 27, no. 1: 180–193. 10.1080/10942912.2023.2301569.

[fsn34516-bib-0017] Islam, M. N. , O. Korner , J. S. Pedersen , J. N. Sorensen , and M. Edelenbos . 2019. “Analyzing Quality and Modelling Mass Loss of Onions During Drying and Storage.” Computers and Electronics in Agriculture 164: 104865. 10.1016/j.compag.2019.104865.

[fsn34516-bib-0018] Javed, M. S. , M. A. Randhawa , Z. Ahmad , M. W. Sajid , M. A. Nasir , and M. R. Tariq . 2017. “Effect of CaCl_2_ and Controlled Atmosphere Storage on Phytochemical Attributes of Guava.” Food Science and Technology 38, no. 2: 356–362. 10.1590/1678-457X.05517.

[fsn34516-bib-0019] Jiang, Y. , and Y. Li . 2001. “Effects of Chitosan Coating on Postharvest Life and Quality of Longan Fruit.” Food Chemistry 73, no. 2: 139–143. 10.1016/s0308-8146(00)00246-6.

[fsn34516-bib-0020] Karmaker, E. , M. Robbani , M. M. Islam , M. A. Suem , and L. A. Labib . 2024. “Elevated Salt Stress Level Affected the Productivity and Chlorophyll Content of *Centella asiatica* (L.).” Archives of Agriculture and Environmental Science 9, no. 2: 324–328. 10.26832/24566632.2024.0902017.

[fsn34516-bib-0021] Kushwaha, R. , M. Singh , V. Singh , V. Puranik , and D. Kaur . 2021. “Variation of Bioactive Compounds and Antioxidant Activity During Ripening of Tomato Cultivars.” Journal of Food and Agriculture Research 1, no. 1: 15–29.

[fsn34516-bib-0022] Kuwar, U. , S. Sharma , and V. R. R. Tadapaneni . 2015. “Aloe Vera Gel and Honey‐Based Edible Coatings Combined With Chemical Dip as a Safe Means for Quality Maintenance and Shelf Life Extension of Fresh‐Cut Papaya.” Journal of Food Quality 38, no. 5: 347–358. 10.1111/JFQ.12150.

[fsn34516-bib-0023] Lane, J. H. , and L. Eynon . 1923. “Methods for Determination of Reducing and Non‐reducing Sugars.” Journal of Sciences 42: 32–37.

[fsn34516-bib-0024] Maftoonazad, N. , and H. S. Ramaswamy . 2005. “Postharvest Shelf‐Life Extension of Avocados Using Methyl Cellulose‐Based Coating.” LWT ‐ Food Science and Technology 38, no. 6: 617–624. 10.1016/j.lwt.2004.08.007.

[fsn34516-bib-0025] Martinez‐Espla, A. , P. J. Zapata , D. Valero , D. Martínez‐Romero , H. M. Díaz‐Mula , and M. Serrano . 2018. “Preharvest Treatments With Salicylates Enhance Nutrient and Antioxidant Compounds in Plum at Harvest and After Storage.” Journal of the Science of Food and Agriculture 98, no. 7: 2742–2750. 10.1002/JSFA.8770. 29105771

[fsn34516-bib-0026] Mditshwa, A. , L. S. Magwaza , S. Z. Tesfay , and U. L. Opara . 2017. “Postharvest Factors Affecting Vitamin C Content of Citrus Fruits: A Review.” Scientia Horticulturae 218: 95–104. 10.1016/J.SCIENTA.2017.02.024.

[fsn34516-bib-0027] Mishra, V. , G. S. Abrol , and N. Dubey . 2018. “Sodium and Calcium Hypochlorite as Postharvest Disinfectants for Fruits and Vegetables.” In Postharvest Disinfection of Fruits and Vegetables, 253–272. Elsevier. 10.1016/B978-0-12-812698-1.00014-5.

[fsn34516-bib-0028] Nasution, Z. , J. N. W. Ye , and Y. Hamzah . 2015. “Characteristics of Fresh‐Cut Guava Coated With *Aloe Vera* Gel as Affected by Different Additives.” Agriculture and Natural Resources 49, no. 1: 111–121. https://li01.tci‐thaijo.org/index.php/anres/article/view/243523.

[fsn34516-bib-0029] Palou, L. , A. Marcilla , C. Rojas‐Argudo , M. Alonso , J. A. Jacas , and M. Á. del Río . 2007. “Effects of X‐Ray Irradiation and Sodium Carbonate Treatments on Postharvest Penicillium Decay and Quality Attributes of Clementine Mandarins.” Postharvest Biology and Technology 46, no. 3: 252–261. 10.1016/J.POSTHARVBIO.2007.05.006.

[fsn34516-bib-0030] Paniagua, C. , P. Ric‐Varas , J. A. Garcia‐Gago , et al. 2020. “Elucidating the Role of Polygalacturonase Genes in Strawberry Fruit Softening.” Journal of Experimental Botany 71, no. 22: 7103–7117. 10.1093/jxb/eraa398. 32856699

[fsn34516-bib-0031] Paul, D. , J. Howlader , M. R. Akon , and L. A. Labib . 2023. “Effect of Hydrocooling and Storage Condition on Postharvest Quality of Coriander Leaf.” International Journal of Innovative Research 8, no. 3: 63–71. http://www.irsbd.org/papers/3._8(3)_December_2023__.pdf.

[fsn34516-bib-0033] Perera, C. O. , and B. Smith . 2013. “Technology of Processing of Horticultural Crops.” In Handbook of Farm, Dairy and Food Machinery Engineering: Second Edition, 259–315. Elsevier. 10.1016/B978-0-12-385881-8.00011-2.

[fsn34516-bib-0034] Perez‐Vazquez, A. , P. Barciela , M. Carpena , and M. A. Prieto . 2023. “Edible Coatings as a Natural Packaging System to Improve Fruit and Vegetable Shelf Life and Quality.” Foods 12, no. 19: 3570. 10.3390/FOODS12193570. 37835222 PMC10572534

[fsn34516-bib-0035] Pietrosanto, A. , C. Leneveu‐Jenvrin , L. Incarnato , P. Scarfato , and F. Remize . 2022. “Antimicrobial, Sealable and Biodegradable Packaging to Maintain the Quality of Shredded Carrots and Pineapple Juice During Storage.” Journal of Food Science and Technology 59, no. 8: 3139–3149. 10.1007/S13197-022-05435-Y. 35872716 PMC9304463

[fsn34516-bib-0047] Ramachandra C. T. , and P. S. Rao . 2008. “Processing of Aloe Vera Leaf Gel.” American Journal of Agricultural and Biological Sciences 3, no. 2: 502–510. 10.3844/ajabssp.2008.502.

[fsn34516-bib-0036] Ranganna, S. 1977. Manual of Analysis of Fruit and Vegetable Products. New Delhi: Tata McGraw‐Hill Publishing Company Limited.

[fsn34516-bib-0037] Ranganna, S. 1986. Handbook of Analysis and Quality Control for Fruits and Vegetables Products. New Delhi: Tata McGraw‐Hill Publishing Company Limited.

[fsn34516-bib-0038] Serna‐Escolano, V. , M. Gutierrez‐Pozo , A. Dobon‐Suarez , P. J. Zapata , and M. J. Gimenez . 2023. “Effect of Preharvest Treatments With Sodium Bicarbonate and Potassium Silicate in Navel and Valencia Oranges to Control Fungal Decay and Maintain Quality Traits During Cold Storage.” Agronomy 13: 2925. 10.3390/agronomy13122925.

[fsn34516-bib-0039] Serrano, M. , J. M. Valverde , F. Guillen , S. Castillo , D. Martinez‐Romero , and D. Valero . 2006. “Use of *Aloe Vera* Gel Coating Preserves the Functional Properties of Table Grapes.” Journal of Agricultural and Food Chemistry 54, no. 11: 3882–3886. 10.1021/jf060168p. 16719510

[fsn34516-bib-0040] Sims, D. A. , and J. A. Gamon . 2002. “Relationships Between Leaf Pigment Content and Spectral Reflectance Across a Wide Range of Species, Leaf Structure and Developmental Stages.” Remote Sensing of Environment 81: 337–354.

[fsn34516-bib-0041] Tang, R. , Y. He , and K. Fan . 2023. “Recent Advances in Stability Improvement of Anthocyanins by Efficient Methods and Its Application in Food Intelligent Packaging: A Review.” Food Bioscience 56: 103164. 10.1016/J.FBIO.2023.103164.

[fsn34516-bib-0042] Ullah, A. , N. A. Abbasi , M. Shafique , and A. A. Qureshi . 2017. “Influence of Edible Coatings on Biochemical Fruit Quality and Storage Life of Bell Pepper cv. “Yolo Wonder”.” Journal of Food Quality 2017: 2142409, 11 pages. 10.1155/2017/2142409.

[fsn34516-bib-0043] Wang, L. , Y. Guo , X. Wang , and X. Zhang . 2022. “Short‐Term O_2_/CO_2_ Controlled Atmosphere Altered the Water Status and Thus Promoted Phenolic Biosynthesis During Wound Healing of Fresh‐Cut White Mushroom (*Agaricus bisporus*).” Postharvest Biology and Technology 188: 111879. 10.1016/j.postharvbio.2022.111879.

[fsn34516-bib-0044] Wu, J. , L. Zhang , and K. Fan . 2022. “Recent Advances in Polysaccharide‐Based Edible Coatings for Preservation of Fruits and Vegetables: A Review.” Critical Reviews in Food Science and Nutrition 64, no. 12: 3823–3838. 10.1080/10408398.2022.2136136. 36263979

[fsn34516-bib-0045] Yearbook of Agricultural Statistics‐2023 (Vol. 35) . 2024. “Bangladesh Bureau of Statistics (BBS) Statistics and Informatics Division (SID), Ministry of Planning Government of the People's Republic of Bangladesh.”

[fsn34516-bib-0046] Youssef, K. , and S. R. Roberto . 2014. “Applications of Salt Solutions Before and After Harvest Affect the Quality and Incidence of Postharvest Gray Mold of ‘Italia’ Table Grapes.” Postharvest Biology and Technology 87: 95–102. 10.1016/J.POSTHARVBIO.2013.08.011.

